# A historical review of 'phossy jaw'

**DOI:** 10.1038/s41415-023-5859-9

**Published:** 2023-06-09

**Authors:** Hugh Devlin

**Affiliations:** Professor of Restorative Dentistry, University of Manchester, Faculty of Biology, Medicine and Health, Division of Dentistry, Room G1 125, Coupland Building 3, Oxford Road, Manchester, M13 9PL, United Kingdom

## Abstract

The aim of this article is to stimulate interest and discussion on the pathogenesis of 'phossy jaw'. Historical evidence from newspapers and articles of the time is presented, as other scientific evidence is largely absent. It has stimulated considerable interest in present-day media due to the struggles of nineteenth century reformers to improve working conditions against an apathetic government and weak enforcement of regulation. Those afflicted were often young women who suffered severe pain, loss of segments of jaw, and disfigurement.

## Key points

- Conditions in the factories where matches were manufactured in the late nineteenth century were harsh and far short of modern standards.
- The factory conditions led some workers to develop 'phossy jaw', a painful and disfiguring condition of unknown pathogenesis.
- The main clinical presentation of 'phossy jaw' included jaw necrosis and a chronic osteomyelitis.

## Introduction

The causative agent of 'phossy jaw' was white phosphorous, which is composed of four phosphorous atoms (P_4_) and is very toxic when inhaled or ingested. It formed an ingredient of 'strike-anywhere' or Lucifer matches in the late nineteenth century. Wooden splints were dipped into a heated mixture to create the matches and then left to dry in a heated chamber. Workers were exposed to the phosphorous vapour or had skin contamination from handling the moist matches.

## Historical context

The first strike of female workers at the Bryant and May match factory began in Bow in the East End of London in 1888. They were protesting against the low pay and very harsh working conditions there. In 1892, it was reported as common practice that wages were stopped for those who went to the hospital to obtain treatment rather than going to the Bryant and May doctor.^1^

By 1892, regulations in Germany stated that the workforce must wash their hands and rinse out their mouths with water before leaving the match factory. In Norway and Sweden, regulations went further and provided for ventilation and size of workrooms, but no such law was available in the UK. Regulation in the UK in 1891 had required manufacturers of phosphorous matches to inform the Medical Officer of Health of any jaw swelling or necrosis. There was insufficiently rigorous enforcement of the Factory Act at the time. Moreland and Sons (match manufacturers) were fined in 1894 for failing to report a case of necrosis.^2^ Bryant and May were also fined for failing to report 17 cases of phosphorous poisoning, despite assurances to the factory inspectors that their workforce was healthy and unaffected.^3^ The fines and costs to Bryant and May amounted to £25.9s, a totally inadequate sum for such a gross offence.

Why was white phosphorous use in matches not banned in the late Victorian era, given its obvious dangers? By 1899, there were alternatives; safety matches were available. These involved the use of the much safer red phosphorous. The main reason was a fear that the match export trade would be 'driven into the hands of competitors working under no restriction'.^4^ However, the same article urged regular dental inspections among workers to avoid contracting disease. A report to Parliament in 1899 stated that 3,134 people were employed in working with phosphorous, with 36 affected with 'phossy jaw' between 1894 and 1897. However, the prevalence among those working in close contact with white phosphorous was probably higher, at about 1.0%.^5^ The Government was reluctant to ban the use of white phosphorous, preferring to prevent disease by legislation for improved working conditions. Some medical opinion agreed: Dr Talbot, Medical Officer for Health at Bow, advocated mixing of the materials on the factory roof with automatic mixers to avoid worker contamination.^6^ However, two cases of phossy jaw were reported at this factory. It was not until 1908 that the White Phosphorous Matches Prohibition Act came into force. This legislation prohibited the manufacture or import of white phosphorous.

## The clinical features of 'phossy jaw'

Those affected by 'phossy jaw' had been in contact with white phosphorous for some years. Most of the affected workers appeared generally well but some underwent a gradual deterioration in health. Newspaper accounts of the 1890s describe a phosphorescent glow from the patient's breath and from the necrotic bone when they were in a darkened room.^7^ Many of the victims had previously suffered from toothache, followed by non-healing of the extraction socket and bone necrosis of the jaws.

There have been some reports that compare medication-related osteonecrosis of the jaw (MRONJ) to 'phossy jaw'.^8^^,^^9^^,^^10^ The clinical presentation of non-healing extraction sockets and bone sequestra are similar in both conditions, but diffuse osteosclerosis is a characteristic feature of MRONJ only and is not associated with 'phossy jaw'.

There are two dried mandible specimens from patients with 'phossy jaw' that are in the Hunterian Museum in London. In one specimen, donated by Charles Gaine, sclerosis is evident in the region immediately adjacent to the necrotic bone but not elsewhere. The other specimen, from a 35-year-old man, is in the collection of Sir John Tomes, and shows anterior cortical resorption but no obvious sclerosis or cortical expansion.

## Radiographic findings in 'phossy jaw'

There are two reports from the 1940s which describe the radiographic findings of 'phossy jaw' in patients who had worked in the manufacture of phosphorous for ten years or more.^11^^,^^12^ A chronic osteomyelitis was present, but the jaw radiographs show no features of diffuse sclerosis. Patients with MRONJ typically have an increased thickness of the mandibular cortex and diffuse sclerosis, narrowing of the mandibular canal and thickening of the lamina dura.

## Discussion

The pathogenesis of 'phossy jaw' is unknown. Hinshaw and DuBose Quin^13^ proposed that pyrophosphoric acid was produced when phosphorous was heated in air, but whether this molecule has any affinity to vital bone was not shown. Delong *et al.*^14^ showed that an infusion of inorganic pyrophosphate can inhibit parathyroid hormone-induced bone resorption in mice. However, the clinical and radiographic evidence of 'phossy jaw' indicates that an inhibition of bone resorption is not the principal aetiological agent. Photographs of the bone sequestra show that they are very irregular, highly resorbed, and porous. They were described as looking like 'clinkers from a coal furnace'.^12^

The major feature of 'phossy jaw is a loss of bone vitality, followed by features of a chronic osteomyelitis. Systemic effects of those affected were rarely reported, indicating that a local application to the mouth of the phosphorous-containing paste by contaminated fingers may be the main cause. The first clinical sign of impending disease was the appearance of a localised, dull red mucosa, followed by ulceration and chronic infection. This would indicate that the point of entry of the phosphorous was by way of the gingival mucosa. This local effect of the phosphorous was limited in time, as some reports described a remarkable regeneration of the mandible during the healing phase, indicating that the mucoperiosteum recovered if damaged.^12^

Due to the lack of modern techniques available to clinicians during this period, the pathogenesis of the disease may remain unknown, but it still deserves investigation and debate.
